# Supplementary material for: Effects of Incorporation of Essential Oils in the Jersey Cow Diet on the Quality of Produced Dairy Products (Milk, Cream, and Colonial Cheese)
Source: Foods. 2025 Aug 11;14(16):2788. doi: 10.3390/foods14162788 (PMC12385980; doi:10.3390/foods14162788)
Supplement: Supplementary file 1 [file foods-14-02788-s001.zip › foods-3752358-supplementary.pdf]

Table A1 – Physicochemical and microbiological parameters of milk and cream in lactating dairy cows produced from Jersey cow milk with (EOB) or without (Control) essential oil addition in the diet (mean values).

Variable	Analyse	Control	EOB [1]	Permissible limit [2]	Legislation
**Physicochemical analysis of milk**	Fat, g/100 g	3.70	3.90	Min. 3.0	BRASIL, 2018
	Protein, g/100 g	3.40	3.53	Min. 2.9	
	Total solids extract, g/100 g	12.51	13.35	Min. 11.4	
	Defatted dry extract, g/100 g	8.80	9.45	Min. 8.4	
	Density, g/mL	1.032	1.033	Between 1.028 and 1.034	
	Acidity, g of lactic acid/100 mL	0.13	0.13	Between 0.14 and 0.18	
	Cryoscopic index, ºC	-0.530	-0.523	Between -0.512 and -0.536	
***Microbiological analyses* of milk**	Total Enterobacteria count, UFC/mL	1	1	Max. 10	BRASIL, 2019
**Physicochemical analysis of cream**	Fat, %	52.66	52.68	Min 45%	BRASIL, 1996; BRASIL, 2022
	Protein, g/100 g	2.12	2.20	1,84 to 3.10	
	Ash, mg/100 g	0.39	0.39	0,30	
	Acidity, g lactic acid/100 g	0.07	0.07	Max. 0.2	
***Microbiological analyses* of cream**	Coagulase-positive *Staphylococcus* count	<1,0x10^1	<1,0x10^1	Max. 10^2	BRASIL, 1996
	Mold and yeast count	3,0x10^1	<1,0x10^1	Max. 10^4	
	Salmonella spp. detection	Absence	Absence	Absence	
	Escherichia coli count	<1,0x10^1	<1,0x10^1	Max. 10	
	Staphylococcal enterotoxin detection	Absence	Absence	Absence	

[1] Essential oil blend composed of eucalyptus oil (*Eucalyptus spp.*; 157.9 g/L), peppermint oil (*Mentha spp.*; 32 g/L), and menthol crystals (55 g/L) (BronchoVest, Biochem, Germany)

[2] According to Brazilian legislation cited in the last column of the table.

Table A2 – Physicochemical and microbiological parameters of colonial cheese in lactating dairy cows produced from Jersey cow milk with or without essential oil addition in the diet (mean values).

Parameter	Maturation time colonial cheese, days						Permissible limit [2]	Legislation
	7		20		45			
	**Control**	**EOB [1]**	**Control**	**EOB [1]**	**Control**	**EOB [1]**		
Moisture, %	47.34	46.74	37.82	34.55	35.61	34.50	Between 35.38 and 81.08	BRASIL, 1996
Fat, g/100 g	28.00	28.50	32.50	35.25	33.50	34.00	Between 25.0 and 44.9	
Protein, g/100 g	19.00	18.91	23.59	23.15	25.07	23.36	Between 10.97 and 27.37	
Ash, mg/100 g	3.65	4.27	4.28	5.06	4.52	5.24	Between 0.84 and 4.32	
pH	5.49	5.53	5.71	6.08	5.78	5.74	Between 4.95 and 6.95	
Mold and yeast	$3,0x10^1$	$1,0x10^1$	$3,0x10^1$	$<1,0x10^1$	$2,5x10^1$	$1,0x10^1$	Max. $5x10^3$	BRASIL, 2022
Coagulase-positive staphylococci	$<1,0x10^1$	$<1,0x10^1$	$<1,0x10^1$	$<1,0x10^1$	$<1,0x10^1$	$<1,0x10^1$	Max. 10^3	
Salmonella spp.	Absence	Absence	Absence	Absence	Absence	Absence	Absence	
Escherichia coli	$<1,0x10^1$	$<1,0x10^1$	$<1,0x10^1$	$<1,0x10^1$	$<1,0x10^1$	$<1,0x10^1$	Max. 10^3	
Staphylococcal enterotoxins	Absence	Absence	Absence	Absence	Absence	Absence	Absence	

[1] Essential oil blend composed of eucalyptus oil (*Eucalyptus spp.*; 157.9 g/L), peppermint oil (*Mentha spp.*; 32 g/L), and menthol crystals (55 g/L) (BronchoVest, Biochem, Germany)

[2] According to Brazilian legislation cited in the last column of the table.
